# Supplementary material for: Protective Effects of Hydrolyzed Chicken Extract (Probeptigen®/Cmi-168) on Memory Retention and Brain Oxidative Stress in Senescence-Accelerated Mice
Source: Nutrients. 2019 Aug 12;11(8):1870. doi: 10.3390/nu11081870 (PMC6722682; doi:10.3390/nu11081870)
Supplement: Supplementary file 1 [file nutrients-11-01870-s001.pdf]

nutrients

Supplementary Materials

Supplementary Table S1: Locomotor activity of 6-month-old SAMP8 mice fed with or without ProBeptigen for 11 weeks in open field test

Sex	Group	Locomotion Time interval (minutes)[2]	
		Initial	Final
Male	Control	106.25±5.67	83.88±5.41
	ProBeptigen (150mg/kg)	120.75±9.12	98.00±5.71
	ProBeptigen (300mg/kg)	110.13±9.61	97.63±3.92
	ProBeptigen (600mg/kg)	112.38±6.60	93.75±5.35
Female	Control	100.25±3.88	97.75±4.13
	ProBeptigen (150mg/kg)	126.38±12.62	117.63±6.14
	ProBeptigen (300mg/kg)	120.50±11.53	112.50±6.21
	ProBeptigen (600mg/kg)	104.63±8.94	101.88±6.38

[1] Values were expressed as mean ± SEM and analyzed by one-way ANOVA. ($n = 8$)

[2] Record time indicated every 5-minutes reading by the monitor E61-21.

Supplementary Table S2: 288 genes (91 genes in male and 197 genes in female SAMP8 mice) were differentially expressed after 13 weeks of ProBeptigen (150 mg/kg) supplementation compared to age-matched control group. Fold change (Ratio of ≥/≤ 2-fold compared with control)

**Male**			**Female**		
**Genes**	**Fold change (ProBeptigen vs Control)**	**p-value**	**Genes**	**Fold change (ProBeptigen vs Control)**	**p-value**
Mup17	19.26	0.0118	Mup1	26.67	0.0039
Mup1	16.45	0.0230	Alb	25.88	0.0085

Mup21	14.26	0.0230	LOC100048884	22.80	0.0030
LOC100048884	12.95	0.0225	Mup17	20.64	0.0021
Mup3	9.24	0.0231	Mup21	19.42	0.0024
Mup19	8.12	0.0340	Alb	17.89	0.0071
Ahsg	7.42	0.0139	Ambp	15.75	0.0016
Alb	7.22	0.0106	Ahsg	15.47	0.0036
Mup-ps16	6.85	0.0348	Mup20	15.09	0.0006
Mup5	5.59	0.0353	Sult2a4	12.95	0.0000
Mup2	5.43	0.0293	Fabp1	12.40	0.0028
Serpina1a	4.65	0.0192	Cyp2b9	12.28	0.0000
Serpina1c	4.56	0.0215	Sult2a2	11.56	0.0001
Serpina1e	3.98	0.0287	Hamp	11.32	0.0001
Nacc1	3.28	0.0039	Sult2a6	10.24	0.0002
Kng1	3.18	0.0115	Cyp2c54	10.09	0.0001
Mup3	2.96	0.0367	Gc	9.74	0.0006
Slc47a1	2.83	0.0041	Ces3a	9.56	0.0001
A130072N09Rik	2.61	0.0337	Apoa5	9.23	0.0014
4921501E09Rik	2.61	0.0034	Cyp2a5	8.25	0.0027
Olfr686	2.39	0.0078	Fgg	8.12	0.0005
Emid1	2.33	0.0044	Mup3	8.09	0.0001
Proz	2.30	0.0073	Wfdc21	7.93	0.0007
S100a9	2.29	0.0003	Kng1	7.75	0.0002
Gm34991	2.22	0.0193	Cyp2b10	7.61	0.0000
Efcab1	2.20	0.0006	Sult2a1	7.60	0.0123
Sval2	2.17	0.0066	Mup2	7.59	0.0006
4930578E11Rik	2.17	0.0275	Cyp2c50	7.40	0.0002
Abhd4	2.09	0.0310	H2-Q10	7.34	0.0010
Hba-a1	2.07	0.0004	Serpina3k	7.25	0.0002
Fam83a	2.06	0.0065	Mup19	7.24	0.0005
Hba-a2	2.06	0.0004	Mbl2	7.02	0.0004
Alas2	2.06	0.0035	Fgb	6.85	0.0007
Slc7a11	2.03	0.0050	Serpinc1	6.68	0.0063
2610316D01Rik	2.01	0.0023	Pzp	6.43	0.0016
Sh3tc1	2.00	0.0398	Cyp2a12	6.32	0.0020
I830077J02Rik	-2.00	0.0084	Serpina6	6.25	0.0005
C330013J21Rik	-2.02	0.0012	Cyp3a16	6.19	0.0010
Cdkn1a	-2.03	0.0117	Serpina1c	6.08	0.0005
Rora	-2.03	0.0038	Serpina1a	6.08	0.0003
4933438K21Rik	-2.06	0.0243	Fmo3	6.03	0.0001
Aldh16a1	-2.06	0.0006	Mup6	6.02	0.0008

Eif4e2	-2.06	0.0061	Cyp3a44	5.90	0.0000
Tspan18	-2.09	0.0180	Mup-ps16	5.83	0.0004
Dnah10	-2.10	0.0115	Serpina1e	5.76	0.0004
Smg9	-2.12	0.0065	Serpina1d	5.71	0.0002
9030607L02Rik	-2.13	0.0031	F2	5.69	0.0002
Olfr1500	-2.18	0.0023	Cyp2f2	5.58	0.0002
Zdhhc2	-2.18	0.0071	Bhmt	5.49	0.0080
Lamtor2	-2.19	0.0058	H2-Q10	5.48	0.0006
Ddx27	-2.20	0.0064	Cyp2c69	5.44	0.0003
BC051408	-2.24	0.0032	Ces1c	5.37	0.0006
Xdh	-2.30	0.0082	Orm1	5.20	0.0000
Phactr3	-2.31	0.0045	Mup21	5.16	0.0001
Lao1	-2.31	0.0046	Kng1	5.09	0.0008
Dcst2	-2.34	0.0309	Car3	4.97	0.0000
Nanos2	-2.39	0.0050	Apoa2	4.97	0.0003
Etnppl	-2.46	0.0052	Serpina1a	4.51	0.0003
Rai14	-2.47	0.0117	Cyp2e1	4.49	0.0020
Cyp39a1	-2.58	0.0214	Adh1	4.42	0.0071
Spdl1	-2.59	0.0073	Mup5	4.30	0.0001
A330015K06Rik	-2.60	0.0022	Bhmt	4.26	0.0005
Plin4	-2.74	0.0025	Hpd	4.16	0.0008
Rbbp8	-2.82	0.0048	Afm	4.07	0.0035
Hmox1	-2.82	0.0178	Cyp2c68	4.05	0.0003
4930421J07Rik	-2.84	0.0006	Mup4	3.98	0.0001
Gm31365	-2.86	0.0439	Cyp3a41a	3.86	0.0006
Olfr711	-2.94	0.0104	Cyp2e1	3.82	0.0380
Gm9895	-2.98	0.0007	Agxt	3.81	0.0013
Xlr4a	-3.04	0.0034	Fgl1	3.53	0.0072
Fam83d	-3.31	0.0062	Apoh	3.47	0.0005
Itgad	-3.54	0.0015	Apoc3	3.36	0.0001
Xlr4b	-3.62	0.0013	Cyp2a4	3.35	0.0003
Gm12945	-4.73	0.0062	Cyp2c29	3.34	0.0014
Itgad	-4.92	0.0005	1700031L13Rik	3.27	0.0011
			H2-M10.3	3.27	0.0075
			Apof	3.26	0.0007
			Cfb	3.19	0.0008
			LOC105242494	3.13	0.0018
			Akr1c20	3.10	0.0102
			Slco1b2	3.10	0.0055

			Mug1	3.05	0.0048
			Mup20	3.01	0.0006
			Gm32930	2.94	0.0011
			Serpina1e	2.93	0.0002
			Slc47a1	2.87	0.0004
			Hp	2.85	0.0005
			Apoc3	2.85	0.0005
			Ssmem1	2.77	0.0077
			Olfr1180	2.72	0.0003
			Cfi	2.67	0.0021
			Olfr661	2.57	0.0047
			Akr1c6	2.57	0.0030
			Serpina1b	2.56	0.0002
			Hpx	2.54	0.0008
			Fgfbp1	2.54	0.0062
			Fgfbp1	2.48	0.0145
			Ifi202b	2.47	0.0242
			Itih4	2.46	0.0007
			9330111N05Rik	2.44	0.0141
			Cyp2d26	2.40	0.0010
			1700021F07Rik	2.39	0.0006
			Olfr979	2.39	0.0007
			1010001N08Rik	2.37	0.0009
			Pkd1l2	2.35	0.0050
			Olfr1260	2.33	0.0017
			Olfr100	2.30	0.0024
			Pld5	2.30	0.0017
			Tex35	2.30	0.0004
			Cmah	2.29	0.0048
			Gm39910	2.29	0.0008
			Rgn	2.29	0.0003
			Ano7	2.28	0.0029
			S100a9	2.28	0.0441
			Gm16702	2.26	0.0001
			4930456L15Rik	2.24	0.0098
			Uox	2.24	0.0003
			Gm28382	2.22	0.0000
			Upb1	2.21	0.0021

			Olfr429	2.21	0.0041
			Mup3	2.19	0.0022
			Alpi	2.18	0.0013
			Angptl3	2.18	0.0005
			Wfdc9	2.16	0.0007
			Gpr20	2.16	0.0274
			4930455J16Rik	2.15	0.0028
			Pdzk1ip1	2.15	0.0037
			Vmn1r59	2.15	0.0087
			4930442E04Rik	2.14	0.0496
			Tspan32	2.13	0.0401
			C3	2.13	0.0002
			Gm3289	2.13	0.0043
			Zic4	2.13	0.0097
			Platr22	2.12	0.0109
			Olfr1062	2.12	0.0164
			Gm17019	2.12	0.0015
			Lox	2.12	0.0000
			Ugt2b37	2.11	0.0044
			Olfr312	2.11	0.0033
			Olfr483	2.10	0.0093
			Slc6a13	2.10	0.0281
			Gm34208	2.10	0.0004
			4933406K04Rik	2.10	0.0049
			Olfr1495	2.09	0.0103
			Mx2	2.08	0.0080
			Sp7	2.07	0.0006
			D630008O14Rik	2.07	0.0011
			Prok2	2.07	0.0186
			Npy4r	2.07	0.0111
			Col1a1	2.06	0.0005
			4930590J08Rik	2.06	0.0001
			2410012M07Rik	2.05	0.0002
			Olfr954	2.05	0.0004
			Wnt10b	2.04	0.0006
			Cyp4a14	2.04	0.0061

			Ptgdr	2.04	0.0050
			Rtp3	2.03	0.0008
			Gm30732	2.03	0.0103
			2010320O07Rik	2.03	0.0263
			Obox1	2.03	0.0081
			Egflam	2.03	0.0001
			Ms4a8a	2.03	0.0154
			Spon2	2.02	0.0278
			Astl	2.02	0.0011
			Hoxa6	2.01	0.0069
			Pla2g6	2.01	0.0005
			Cyp2c37	2.01	0.0018
			Proz	2.01	0.0005
			E030025P04Rik	2.00	0.0014
			Gm33277	-2.01	0.0167
			Gm5907	-2.01	0.0011
			Lpar5	-2.01	0.0007
			Gm10825	-2.02	0.0017
			Nlrp1a	-2.05	0.0006
			Birc3	-2.07	0.0015
			Psg27	-2.07	0.0400
			AA474331	-2.07	0.0059
			Glb1l2	-2.07	0.0001
			Pgk2	-2.07	0.0038
			Olfr586	-2.07	0.0037
			Fev	-2.08	0.0206
			Skint3	-2.11	0.0089
			Olfr136	-2.17	0.0047
			Gm14478	-2.20	0.0111
			Gm9908	-2.24	0.0017
			Olfr1311	-2.24	0.0287
			Traf6	-2.32	0.0424
			4930591A17Rik	-2.33	0.0011
			Olfr1054	-2.36	0.0054
			Skint8	-2.38	0.0076
			LOC102639958	-2.39	0.0201
			4930449C09Rik	-2.39	0.0321

			Olfr494	-2.45	0.0297
			Bid	-2.52	0.0271
			Olfr642	-2.61	0.0001
			Gm31137	-3.12	0.0065
			Olfr794	-3.13	0.0059

© 2019 by the authors. Submitted for possible open access publication under the terms and conditions of the Creative Commons Attribution (CC BY) license (http://creativecommons.org/licenses/by/4.0/).
